# Does social participation decrease the risk of frailty? Impacts of diversity in frequency and types of social participation on frailty in middle-aged and older populations

**DOI:** 10.1186/s12877-022-03219-9

**Published:** 2022-07-02

**Authors:** Ju Sun, Xuying Kong, Haomiao Li, Jiangyun Chen, Qiang Yao, Hanxuan Li, Feng Zhou, Hua Hu

**Affiliations:** 1 School of Political Science and Public Administration, Wuhan University, Wuhan, China; 2 School of Health Management, Southern Medical University, Guangzhou, China; 3 School of Public Health, Wuhan University of Science and Technology, Wuhan, China; 4 College of Medicine and Health Science, Wuhan Polytechnic University, Wuhan, China

**Keywords:** Frailty, Social participation, Frequency, Communicative activities, Intellectually demanding/engaging activities, Community-organized physical activities

## Abstract

**Background:**

Social participation (SP) may be an effective measure for decreasing frailty risks. This study investigated whether frequency and type of SP is associated with decreased frailty risk among Chinese middle-aged and older populations.

**Methods:**

Data were derived from the China Health and Retirement Longitudinal Study (CHARLS). Frailty was assessed using the Rockwood’s Cumulative Deficit Frailty Index. SP was measured according to frequency (none, occasional, weekly and daily) and type (interacting with friends [IWF]; playing mah-jong, chess, and cards or visiting community clubs [MCCC], going to community-organized dancing, fitness, qigong and so on [DFQ]; participating in community-related organizations [CRO]; voluntary or charitable work [VOC]; using the Internet [INT]). Smooth curves were used to describe the trend for frailty scores across survey waves. The fixed-effect model (*N* = 9,422) was applied to explore the association between the frequency/type of SP and frailty level. For baseline non-frail respondents (*N* = 6,073), the time-varying Cox regression model was used to calculate relative risk of frailty in different SP groups.

**Results:**

Weekly (β =  − 0.006; 95%CI: [− 0.009, − 0.003]) and daily (β =  − 0.009; 95% CI: [− 0.012, − 0.007]) SP is associated with lower frailty scores using the fixed-effect models. Time-varying Cox regressions present lower risks of frailty in daily SP group (HR = 0.76; 95% CI: [0.69, 0.84]). SP types that can significantly decrease frailty risk include IWF, MCCC and DFQ. Daily IWF and daily DFQ decreases frailty risk in those aged < 65 years, female and urban respondents, but not in those aged ≥ 65 years, male and rural respondents. The impact of daily MCCC is significant in all subgroups, whereas that of lower-frequent MCCC is not significant in those aged ≥ 65 years, male and rural respondents.

**Conclusion:**

This study demonstrated that enhancing participation in social activities could decrease frailty risk among middle-aged and older populations, especially communicative activities, intellectually demanding/engaging activities and community-organized physical activities. The results suggested very accurate, operable, and valuable intervening measures for promoting healthy ageing.

**Supplementary Information:**

The online version contains supplementary material available at 10.1186/s12877-022-03219-9.

## Background

Frailty describes the vulnerability of an individual to the poor resolution of homeostasis in response to endogenous and exogenous stressors after a sudden change in health status. It denotes a transition phase between healthy ageing and disability, which is undoubtedly an emerging global health burden [1, 2]. Frailty is associated with the independence, physical function, and cognition of an individual, which consequently exposes the individual to a high risk of negative health-related outcomes [3, 4]. Population ageing is rapidly accelerating worldwide, which leads to great challenges associated with the economy, medicine, and society [5]. Thus, ameliorating frailty to promote healthy ageing is a critical and urgent initiative around the world.

Social participation (SP) may be an effective measure for decreasing frailty risks. The continuity theory holds that older people have health barriers due to aging, but alternative activities such as volunteer service and physical training can continue to help them reduce the health damage caused by aging [6, 7]. Previous studies provided evidence that social frailty or social isolation is associated with physical functioning, cognition, and depression, and predicts mortality [8–10]. Social frailty can be defined as the absence of social resources, social activities, and self-management abilities considered important for fulfilling the basic social needs [11, 12]. Moreover, social isolation is typically defined as having less or infrequent social contacts [13]. SP has been demonstrated effective in preventing social frailty/isolation [14], in increasing happiness [15], physical, and cognitive functioning [16, 17], and in reducing the risk of disability in activities or instrumental activities of daily living [18] among older adults. In addition, some studies indicated that individuals with more SP can expand their social network, improve their perception of social cohesion, gain a stronger sense of trust and reciprocity, therefore leading to healthier outcomes [19, 20]. At the same time, they can perceive the strength of social support and improve their own level of social support [21]. To sum up, SP is effective in improving the older population’s physical and psychological health status, and has a potential to prevent frailty, or reduce existing frailty. However, research that directly proves the impact of SP on frailty is scarce.

With the improvement in living standards, SP has become a component of the daily life of many residents, whereas an increasing number of types of SP have emerged. SP with different frequencies and types may generate varying levels of effectiveness. For example, Wang [22] argued that transitioning from no SP to one or more types of SP or to a once a week or higher frequency was associated with a decline in depressive symptoms. An observational study conducted in Japan demonstrates that exercise-based social participation has associations with reversing frailty progression [23]. SP is one of the most effective interventions with relatively low costs of resources, and can be promoted through advocacy, education, and community activities. Therefore, exploring the association between SP and frailty, and identifying the most effective type and frequency of SP can be a promising initiative for promoting healthy ageing.

This study aims to explore the effect of SP on frailty among older populations by comparing its impacts according to the frequency and type of SP. Moreover, this study hypothesizes that high-frequency SP is associated with less risk of frailty, whereas the impact differs among the types of SP.

## Methods

### Data sources

Data were derived from the China Health and Retirement Longitudinal Study (CHARLS) for 2011, 2013, 2015, and 2018. CHARLS was conducted by the National School of Development of Peking University, which collected high-quality microdata from middle-aged and older individuals associated with health status, demographics, and economic information in China. Data are nationally representative of China due to the use of multistage stratified probability-proportionate-to-size sampling. A detailed description of the objectives and methods of CHARLS has been reported previously [24].

**Fig. 1:**
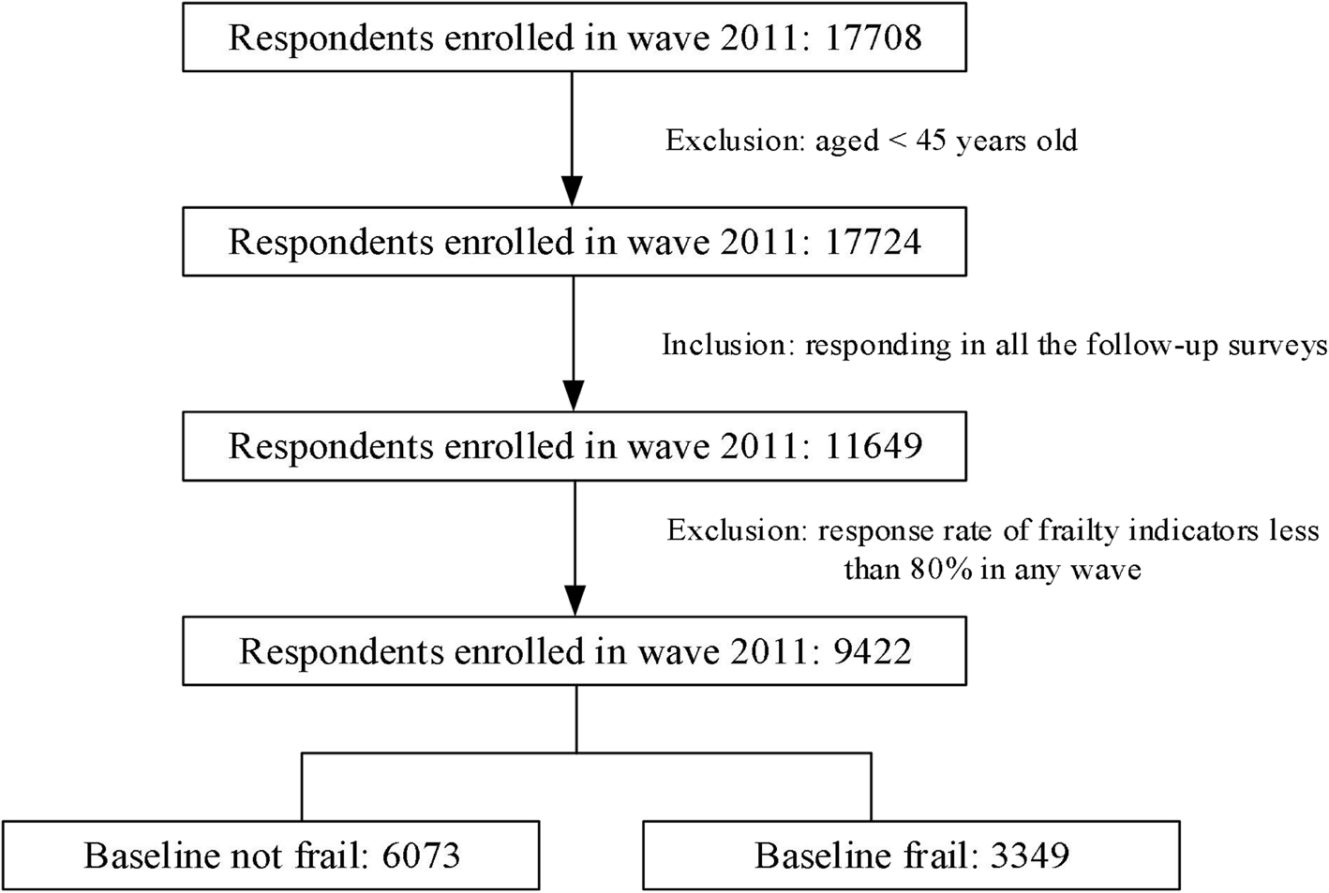
Flow chart of sample selection

The current study included data from participants who were interviewed at baseline (2011), aged 45 years and older as of 2011, and remained for the following waves. Participants with missing values for the dependent or independent variables were excluded. Figure 1 presents the details of sample selection.

### Variables

#### Frailty

The outcome of this study is frailty, which is defined following Rockwood's Cumulative Deficit Frailty Index (FI) [25]. As suggested by Moorhouse & Rockwood, the FI allows inclusion of any health deficit providing that a minimum of 30 deficits in total are included and that each deficit is associated with adverse health outcomes; increases in prevalence with age at least into the tenth decade; has a prevalence of at least 1% in the population; and does not saturate [26]. A total of 54 items were selected to calculate FI in this study, including physical limitations, psychological symptoms, comorbidities, history of trauma, cognitive deficits [27, 28]. All variables were coded as 0 or 1, with details shown in Supplementary Table S1. The frailty index was calculated by summing the number of deficits reported by the respondents and dividing it by the total number of answered possible deficits. For the chronic disease variables (e.g., hypertension), a score of 1 in one wave was allocated to all subsequent waves, because these conditions are irreversible. A frailty index with potential ranging from 0 to 1 was generated. High scores indicate high levels of frailty. Given the accuracy of the frailty index, individuals with a denominator of less than 43 (80% out of the total 54 items) were excluded from the study [7, 29]. When analyzing the risk of frailty by hazard ratio, frailty was categorized using a defined cutoff point of 0.25 according to previous studies (i.e., non-frail or prefrail: < 0.25; frail: ≥ 0.25–1.00) [30, 31].

### Social participation

SP is the exposure variable in this study. SP in CHARLS included (1) interacting with friends (IWF), (2) playing mah-jong, chess, and cards or visiting community clubs (MCCC), (3) going to (community-organized) dancing, fitness, Chinese Qigong (a system of deep breathing exercises) and so on (DFQ), (4) participating in community-related organizations (CRO), (5) undertaking voluntary or charitable work, (6) caring for a distant sick or disabled adult, (7) providing help to relatives, friends, or neighbors, and (8) using the Internet (INT). Types 5–7 were categorized into one group as *voluntary or charitable work* (VOC) [22, 32]. Frequency of each type of SP was categorized into four groups, namely, (1) none, (2) occasional, (3) weekly, and (4) nearly daily. The frequency of the comprehensive SP in each respondent was defined as his/her highest frequency of the above SP types. Supplementary Table S2 indicates the distribution of SP types. The frequencies of IWF and MCCC are higher than those of other SP types.

### Covariates

The covariates were first identified within the literature. Then we explored their association with the independent variable and their impact on the change of the association between the independent variable and dependent variable. Covariates were included in the main analysis as potential confounders if they changed the estimates of the effect of SP on frailty by more than 10% or were significantly associated with the frailty score [33]. Confounders were selected based on a generalized estimating equation, as the data were repeated measurements. The final covariates included: age, gender, marital status, hukou status (which is a special identifier in China and affects many aspects of life in China such as buying a house, buying a car, children’s school enrollment and other welfare) [34], level of education, rural/urban residence, public health insurance coverage, current work status [35], alcohol intake, smoking status, and household per capita consumption (which is a a proxy for socioeconomic status) [36]. Supplementary Table S3 presents the definitions and grouping details for the covariates.

### Statistical analysis

In description analysis of the respondents’ baseline characteristics within different SP groups, "number (percentage)" and "mean ± standard deviation (SD) " were used for the description of binary or categorical variables and continuous variables, respectively.

To observe the trajectory of frailty level along with time, we adopted the general additive models (GAM) to fit the regression for frailty on survey wave with identity used as link functions for the outcomes and all the covariates adjusted. GAM extends the generalized linear model, in which the predictor function may contain one or more user-specified sums of smooth functions of the covariates plus a conventional parametric component of the linear predictor [37]. That means, with the cubic spline smoothing function to control for the confounding factors, an additional smoothing function of survey wave could be constructed to filter out the trajectory of frailty level [38, 39]. In addition, the curve could reveal the trajectory variance between different groups. Therefore, we also compared the trajectory variance between those frail at baseline and non-frail at baseline.

Moreover, this study employed a longitudinal linear fixed-effect regression model to estimate the association between SP frequency, SP type, and frailty scores across the four waves. F-test and Hausman test were employed for model selection among ordinary least squares (OLS), random-effects model, and fixed-effects model. The F-test between the pooled ordinary least squares (OLS) and fixed-effect models yielded statistical significance (*P* < 0.001), which indicated that the the fixed-effect model was prior to the OLS model. A Hausman specification test was then conducted between the fixed- and the random-effect models, which also yielded statistical significance (*P* < 0.001), and indicated that the fixed-effect model was prior to the random-effect model [40]. The fixed effects model treats each individual as their own control and has the advantage of reducing biases brought about by between-individual and hard-to-observe factors [22].

The Cox proportional hazard models were further performed to calculate relative risk with survey waves as the timescale. However, SP conditions may vary across waves; thus, we were unable to roughly define exposure, which may lead to immortal time bias [41]. Therefore, we performed the Cox regression with time-varying exposure and covariates to avoid this bias [42]. HRs with 95% CIs were calculated. The respondents classified as frail at baseline were excluded from analysis, whereas those who remained non-frail as of 2018 were considered censored data. When analyzing SP types, fixed-effects models and time-varying Cox regression models were also performed to explore the associations between frequency of each SP type and frailty risk, with all the selected covariates controlled.

To avoid statistical test performance reduction and bias due to the direct exclusion of missing values, multiple imputations (MIs) were conducted to impute the missing covariate values at baseline based on five replications and a chained equation approach. Supplementary Table S4 provides the number of missing values and MI evaluation. The missing values in the subsequent waves were then imputed using baseline data, because the majority of the conditions were stable or changed little.

To validate the results, two sensitivity analyses were conducted. First, we used the data without imputation to repeat the fixed-effect and Cox analyses. Second, some studies suggested the deficit to calculate frailty index should have a missingness of 5.0% or less [43, 44]. Therefore, we identified participants who responded to 52 or more items on frailty-related deficits and repeated the analysis. Furthermore, as individuals may experience worsened or improved frailty state over time, we identified respondents who were categorized under the frailty group at baseline and set frailty improvement as the outcome variable to explore the association between SP and improvement in frailty.

In addition, we performed several subgroup analyses to identify the associations among specific respondents, including (1) those aged ≥ 65 years versus those aged < 65 years; (2) male versus female participants; and (3) rural versus urban participants.

We did not account for sampling and non-response weight in the analysis, because many studies that employed the CHARLS data suggested that the results of regression analyses with and without weighting were similar [36]. *P* values were two-tailed, where statistical significance was set at an alpha level of 0.05. Data were analyzed using Stata (version 15) and R version 3.6.3 (R Foundation for Statistical Computing, Vienna, Austria).

### Results

**Fig. 2:**
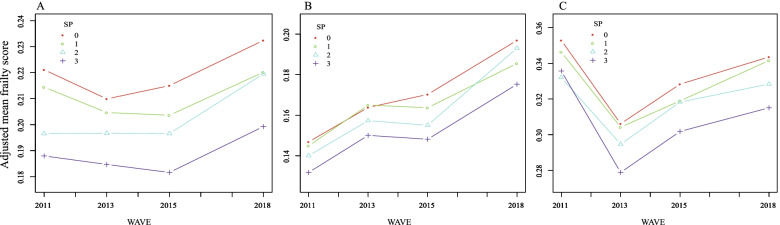
Smoothing curves fitting for the dynamic changes in frailty scores across the 4 survey waves. **A**. Smoothing curves fitting for the change of mean frailty scores within different SP groups. **B.** Smoothing curves fitting for the change of mean frailty scores within different SP groups among respondents not frail at baseline. **C.** Smoothing curves fitting for the change of mean frailty scores within different SP groups among respondents frail at baseline. (0 = non-SP; 1 = occasional SP; 2 = weekly SP; 3 = daily SP. Smoothing curves were constructed based on general additive models, with all the covariates adjusted.)

Out of the 17,708 participants in the baseline, we included 9,422 respondents in the analysis (Fig. 1). Among them, 4,667, 1,411, 1,101, and 2,243 respondents were grouped under non-SP, occasionally, weekly, and daily, respectively (Table 1). Participants under the non-SP and daily groups are older than the other two groups and are composed of more women. The mean frailty score increased from 0.22 in 2011 to 0.28 in 2018, whereas the prevalence of frailty increased from 35.54% to 53.50% correspondingly (Supplementary Table S5). Figure 2 reveals the dynamic changes in frailty scores across the survey waves. After adjusting for all covariates, we observed a remarkable variance in the trends within groups, where the adjusted mean scores for the daily group were lower than those for three other groups were (Fig. 2A). For the baseline non-frail respondents (Fig. 2B), the frailty score for the daily SP group was also the lowest. Moreover, we observed a remarkable intersection between occasional and weekly SP from 2015 to 2018, after which the frailty score for weekly SP exceeded that of occasional SP. As for respondents who were frail at baseline (Fig. 2C), the daily SP group continued to score lowest infrailty.

**Table 1:** Baseline description of the sample within different SP frequency groups*

	All	Non-SP	Occasional	Weekly	Daily
Number of participants	9422	4667	1411	1101	2243
Age	57.68 ± 8.24	58.06 ± 8.05	56.44 ± 7.94	56.72 ± 8.34	58.17 ± 8.64
Gender					
Male	4362 (46.30%)	2130 (45.64%)	714 (50.60%)	586 (53.22%)	932 (41.55%)
Female	5060 (53.70%)	2537 (54.36%)	697 (49.40%)	515 (46.78%)	1311 (58.45%)
Education levels					
Less than lower secondary	8362 (88.75%)	4298 (92.09%)	1239 (87.81%)	934 (84.83%)	1891 (84.31%)
Upper secondary & vocational training	933 (9.90%)	344 (7.37%)	154 (10.91%)	146 (13.26%)	289 (12.88%)
Tertiary	127 (1.35%)	25 (0.54%)	18 (1.28%)	21 (1.91%)	63 (2.81%)
Marital status					
Divorced or widowed	951 (10.09%)	476 (10.20%)	120 (8.50%)	89 (8.08%)	266 (11.86%)
Married	8471 (89.91%)	4191 (89.80%)	1291 (91.50%)	1012 (91.92%)	1977 (88.14%)
Hukou status					
Agricultural	7711 (81.85%)	4009 (85.92%)	1182 (83.77%)	880 (79.93%)	1640 (73.12%)
Non-agricultural	1647 (17.48%)	625 (13.39%)	223 (15.80%)	213 (19.35%)	586 (26.13%)
Other	63 (0.67%)	32 (0.69%)	6 (0.43%)	8 (0.73%)	17 (0.76%)
Rural/urban residence					
Rural	6153 (65.30%)	3160 (67.71%)	974 (69.03%)	717 (65.12%)	1302 (58.05%)
Urban	3269 (34.70%)	1507 (32.29%)	437 (30.97%)	384 (34.88%)	941 (41.95%)
Morbidity					
None	2843 (30.17%)	1397 (29.93%)	431 (30.55%)	337 (30.61%)	678 (30.23%)
Single	2805 (29.77%)	1419 (30.40%)	442 (31.33%)	309 (28.07%)	635 (28.31%)
Morbidity	3774 (40.06%)	1851 (39.66%)	538 (38.13%)	455 (41.33%)	930 (41.46%)
Public health insurance coverage					
Not covered	566 (6.02%)	289 (6.21%)	69 (4.90%)	57 (5.18%)	151 (6.76%)
Covered	8834 (93.98%)	4367 (93.79%)	1340 (95.10%)	1043 (94.82%)	2084 (93.24%)
Current work status					
Not working	2319 (24.68%)	1036 (22.27%)	252 (17.89%)	242 (22.04%)	789 (35.25%)
Working	7078 (75.32%)	3616 (77.73%)	1157 (82.11%)	856 (77.96%)	1449 (64.75%)
Alcohol intake					
Do not drink	6308 (66.95%)	3246 (69.55%)	858 (60.81%)	668 (60.67%)	1536 (68.48%)
Drink	3114 (33.05%)	1421 (30.45%)	553 (39.19%)	433 (39.33%)	707 (31.52%)
Smoking status					
Never	5810 (61.67%)	2939 (62.99%)	794 (56.27%)	620 (56.31%)	1457 (64.96%)
Quit now	752 (7.98%)	362 (7.76%)	124 (8.79%)	104 (9.45%)	162 (7.22%)
Smoke	2859 (30.35%)	1365 (29.25%)	493 (34.94%)	377 (34.24%)	624 (27.82%)
Household per capita consumption					
Low	3178 (39.28%)	1699 (42.71%)	489 (39.79%)	344 (36.13%)	646 (33.45%)
Low to middle	2369 (29.28%)	1192 (29.96%)	363 (29.54%)	259 (27.21%)	555 (28.74%)
Middle	1706 (21.09%)	767 (19.28%)	260 (21.16%)	230 (24.16%)	449 (23.25%)
High	837 (10.35%)	320 (8.04%)	117 (9.52%)	119 (12.50%)	281 (14.55%)

*SP* Social participation

^*^Mean ± standard deviation was used to describe continuous variables, and number (constituent ratio [%]) was used to describe categorical variables

The fixed-effect model indicates the positive effect of weekly (β =  − 0.006; 95% CI: [− 0.009, − 0.003]; *P* < 0.001) and daily (β =  − 0.009; 95% CI: [− 0.012, − 0.007]; *P* < 0.001) SP on frailty score compared with those of the non-SP group (Table 2). Data from 6,073 respondents underwent time-varying Cox analysis. Supplementary Table S6 presents the baseline characteristics of these respondents. The Cox regression presents a lower risk of frailty in the daily SP group compared with the non-SP group (HR = 0.76; 95% CI: [0.69, 0.84]; *P* < 0.001). The two models also consistently indicate that age, educational level, marital status, Hukou status, comorbidity and smoking status are risk factors of frailty.

**Table 2:** The association between SP frequency and frailty based on fixed-effects model and time-varying Cox regression

	Fixed-effects model (*n* = 9422)		Time-varying Cox regression (*n* = 6073)	
	Coefficient (95%CI)	*P* value	HR(95%CI)	*P* value
**Frequency of SP (ref. Non SP)**				
Occasional	0.000 (-0.003, 0.002)	0.776	0.93 (0.83, 1.04)	0.187
Weekly	-0.006 (-0.009, -0.003)	< 0.001	0.97 (0.86, 1.09)	0.577
Daily	-0.009 (-0.012, -0.007)	< 0.001	0.76 (0.69, 0.84)	< 0.001
Age	0.002 (0.001, 0.002)	< 0.001	1.01 (1.00, 1.01)	< 0.001
Gender (ref. Male)				
Female	0.019 (-0.043, 0.080)	0.554	1.87 (1.67, 2.10)	< 0.001
Rural/urban residence (ref. Rural)				
Urban^a^	–	–	0.75 (0.68, 0.82)	< 0.001
Education level (ref. Less than lower secondary)				
Upper secondary & vocational training	-0.014 (-0.025, -0.003)	0.015	0.73 (0.64, 0.84)	< 0.001
Tertiary	-0.025 (-0.046, -0.004)	0.020	0.62 (0.42, 0.90)	0.013
Marital status (ref. Divorced or widowed)				
Married	-0.027 (-0.032, -0.021)	< 0.001	0.77 (0.68, 0.86)	< 0.0001
Hukou status (ref. Agricultural)				
Non-agricultural	-0.010 (-0.016, -0.004)	0.002	0.74 (0.66, 0.84)	< 0.001
Other	-0.010 (-0.019, 0.000)	0.044	0.81 (0.56, 1.18)	0.274
Public health insurance coverage (ref. Not covered)				
Covered	0.002 (-0.002, 0.006)	0.441	0.83 (0.69, 1.00)	0.045
Current work status (ref. Not working)				
Working	-0.018 (-0.020, -0.015)	< 0.001	0.94 (0.86, 1.03)	0.194
Comorbidity (ref. None)				
Single	0.030 (0.026, 0.034)	< 0.001	2.10 (1.83, 2.41)	< 0.001
Morbidity	0.072 (0.067, 0.076)	< 0.001	5.03 (4.43, 5.70)	< 0.001
Household per capita consumption group (ref. low)				
Low to middle	0.000 (-0.003, 0.002)	0.890	0.91 (0.81, 1.02)	0.089
Middle	0.008 (0.005, 0.010)	< 0.001	0.93 (0.84, 1.04)	0.232
High	0.014 (0.011, 0.016)	< 0.001	0.97 (0.87, 1.09)	0.612
Alcohol intake (ref. Do not drink)				
Drink	-0.001 (-0.004, 0.002)	0.407	0.96 (0.88, 1.06)	0.434
Smoking status (ref. Never)				
Quit now	0.011 (0.006, 0.016)	< 0.001	1.20 (1.05, 1.38)	0.008
Smoke now	0.000 (-0.005, 0.005)	0.973	1.25 (1.11, 1.40)	< 0.001

*HR* Hazard ratio, *CI* Confidence Interval

^a^The variable “Rural/urban residence” was omitted in the fixed-effects model because of collinearity

According to SP type (Table 3), daily IWF, MCCC and DFQ can significantly decrease the risk of frailty in the fixed-effect model (IWF: β =  − 0.008; 95% CI: [− 0.010, − 0.005], *P* < 0.001; MCCC: β =  − 0.014; 95% CI: [− 0.019, − 0.010], *P* < 0.001; DFQ: β =  − 0.015; 95% CI: [− 0.020 to − 0.010], *P* < 0.001) and the time-varying Cox regression (IWF: HR = 0.89; 95% CI: [0.80, 0.99], *P* = 0.031; MCCC: HR = 0.68; 95% CI: [0.57, 0.80]; *P* < 0.001; DFQ: HR = 0.72; 95% CI: [0.60, 0.86]; *P* < 0.001). For MCCC, occasional frequency was associated with lower frailty risk through the fixed-effect model (β =  − 0.007; 95% CI: [− 0.011, − 0.003], *P* < 0.001) and the Cox model (HR = 0.86; 95% CI: [0.74, 0.98], *P* = 0.028). Weekly MCCC is significantly associated with lower frailty risk through the fixed-effect model (β =  − 0.010; 95% CI: [− 0.014, − 0.006], *P* < 0.001), while this association is almost significant through the Cox model (HR = 0.86; 95% CI: [0.74, 1.00], *P* = 0.056). The weekly DFQ was also associated with decreased frailty risk through the two models (fixed-effect model: β =  − 0.011; 95% CI: [− 0.019, − 0.002], *P* = 0.015; Cox model: HR = 0.59; 95% CI: [0.37, 0.92], *P* = 0.020). The impacts of CRO, VOC and INT on frailty are not significant, except that daily INT is associated with lower frailty risk through fixed-effects model.

**Table 3:** The association of frailty with different SP types based on fixed-effects model and time-varying Cox regression

	Fixed-effects model (*n* = 9422)^a^		Time-varying Cox model (*n* = 6073)^b^	
	Coefficient (95%CI)	*P* value	HR (95%CI)	*P* value
**IWF**				
Occasional	0.002 (-0.001, 0.004)	0.270	0.96 (0.86, 1.08)	0.496
Weekly	-0.004 (-0.008, -0.001)	0.009	0.99 (0.87, 1.13)	0.902
Daily	-0.008 (-0.010, -0.005)	< 0.001	0.89 (0.80, 0.99)	0.031
**MCCC**				
Occasional	-0.007 (-0.011, -0.003)	< 0.001	0.86 (0.74, 0.98)	0.028
Weekly	-0.010 (-0.014, -0.006)	< 0.001	0.86 (0.74, 1.00)	0.056
Daily	-0.014 (-0.019, -0.010)	< 0.001	0.68 (0.57, 0.80)	< 0.001
**DFQ**				
Occasional	-0.008 (-0.016, 0.000)	0.049	0.80 (0.56, 1.13)	0.200
Weekly	-0.011 (-0.019, -0.002)	0.015	0.59 (0.37, 0.92)	0.020
Daily	-0.015 (-0.020, -0.010)	< 0.001	0.72 (0.60, 0.86)	< 0.001
**CRO**				
Occasional	-0.003 (-0.012, 0.005)	0.423	1.04 (0.76, 1.42)	0.823
Weekly	0.000 (-0.012, 0.012)	0.992	0.74 (0.48, 1.16)	0.192
Daily	0.002 (-0.016, 0.020)	0.811	0.55 (0.22, 1.36)	0.193
**VOC**				
Occasional	0.002 (-0.001, 0.005)	0.197	0.97 (0.86, 1.09)	0.641
Weekly	-0.001 (-0.006, 0.005)	0.854	0.97 (0.77, 1.23)	0.824
Daily	0.001 (-0.006, 0.008)	0.823	0.78 (0.57, 1.09)	0.143
**INT**				
Occasional	-0.001 (-0.012, 0.011)	0.910	0.70 (0.42, 1.17)	0.173
Weekly	0.003 (-0.010, 0.016)	0.699	0.57 (0.30, 1.12)	0.102
Daily	0.011 (0.005, 0.017)	< 0.001	0.90 (0.75, 1.08)	0.245

*IWF* Interacting with friends, *MCCC* Playing mah-jong, chess, cards or visiting community clubs, *DFQ* Going to community-organized dancing, fitness, qigong and so on; CRO, participating in community-related organizations, *VOC* Voluntary or charitable work, *INT* Using the Internet, *HR* Hazard ratio, *CI* Confidence Interval

^a“^None” group was set as the reference in each type analysis. Controlled covariates included age, gender, marital status, hukou status, education levels, rural/urban residence, public health insurance coverage, current work status, alcohol intake, smoking status and household per capita consumption

^b^The intensity of each SP type was set as time-variant exposure. ^“^None” group was set as the reference. Age, marital status, hukou status, public health insurance coverage, current work status, alcohol intake, smoking status and household per capita consumption were controlled as time-variant covariates, and gender, education level and rural/urban residence were controlled as fixed covariates

For those categorized as frail at baseline (baseline characteristics are presented in Supplementary Table S7), the time-varying Cox regression (outcome: frailty improvement) also demonstrated a significant association between daily SP and lower frailty risk (HR = 1.39; 95% CI: [1.23, 1.57], *P* < 0.001). For different SP types, weekly and daily IWF, daily MCCC, daily DFQ, daily CRO and occasional INT are effective in improving frailty status. (Table 4).

**Table 4:** The association between frequency and type of SP and frailty based on time-varying Cox regression among respondents frail at baseline (*n* = 3349)*

	**Respondents frail at the baseline**	
	HR (95%CI)	*P* value
**SP**		
Occasional	1.02 (0.88, 1.19)	0.763
Weekly	1.13 (0.95, 1.33)	0.157
Daily	1.39 (1.23, 1.57)	< 0.001
**IWF**		
Occasional	1.01 (0.85, 1.19)	0.941
Weekly	1.27 (1.05, 1.53)	0.012
Daily	1.34 (1.18, 1.53)	< 0.001
**MCCC**		
Occasional	1.08 (0.87, 1.35)	0.476
Weekly	1.20 (0.96, 1.50)	0.116
Daily	1.60 (1.29, 1.98)	< 0.001
**DFQ**		
Occasional	1.39 (0.84, 2.29)	0.198
Weekly	0.90 (0.50, 1.64)	0.733
Daily	1.63 (1.27, 2.08)	< 0.001
**CRO**		
Occasional	1.10 (0.62, 1.94)	0.753
Weekly	1.72 (0.92, 3.22)	0.088
Daily	3.06 (1.27, 7.37)	0.013
**VOC**		
Occasional	0.96 (0.8, 1.15)	0.662
Weekly	0.90 (0.65, 1.23)	0.499
Daily	1.09 (0.72, 1.64)	0.689
**INT**		
Occasional	2.39 (1.11, 5.16)	0.027
Weekly	1.60 (0.56, 4.54)	0.380
Daily	0.87 (0.42, 1.80)	0.713

*SP* Social participation, *IWF* Interacting with friends, *MCCC* Playing mah-jong, chess, cards or visiting community clubs, *DFQ* Going to community-organized dancing, fitness, qigong and so on, *CRO* Participating in community-related organizations, *HR* Hazard ratio, *CI* Confidence Interval

^*^The intensity of each SP type of was set as time-variant exposure. ^“^None” group was set as the reference. Age, marital status, hukou status, public health insurance coverage, current work status, alcohol intake, smoking status and household per capita consumption were controlled as time-variant covariates, and gender, education level and rural/urban residence were controlled as fixed covariates

Additionally, we conducted two sensitivity analyses to validate the results (Table 5). First, we used data without imputation to repeat the fixed-effects and Cox analysis. Second, we included respondents with frailty related items having a missingness of 5.0% or less (at least 52 items). In conclusion, the findings were consistent with those of the previous analysis. In other words, the direction and magnitude of the effects remained consistent, which validated our main analysis.

**Table 5:** Sensitivity analysis*

	Fixed-effects model		Time-varying Cox model	
	Estimate (95%CI)	*P* value	HR (95%CI)	*P* value
**Without imputation**				
Occasional	0.001 (-0.002, 0.005)	0.409	0.93 (0.83, 1.04)	0.186
Weekly	-0.004 (-0.008, -0.001)	0.022	0.97 (0.86, 1.09)	0.593
Daily	-0.009 (-0.012, -0.006)	< 0.001	0.76 (0.70, 0.84)	< 0.001
**Deficit have a missingness of 5.0% or less**				
Occasional	0.000 (-0.004, 0.003)	0.949	0.99 (0.86, 1.13)	0.873
Weekly	-0.007 (-0.011, -0.003)	0.001	0.97 (0.83, 1.14)	0.747
Daily	-0.009 (-0.012, -0.005)	< 0.001	0.83 (0.74, 0.94)	0.002

*HR* Hazard ratio, *CI* Confidence Interval

^*^^“^None” group was set as the reference. Age, marital status, hukou status, public health insurance coverage, current work status, alcohol intake, smoking status and household per capita consumption were controlled as time-variant covariates, and gender, education level and rural/urban residence were controlled as fixed covariates

In the subgroup analysis, we used the time-varying Cox regression to predict risk. Daily SP was associated with decreased risk of frailty in all subgroups. Weekly SP could decrease frailty risk only in urban respondents. We further identified the impacts of IWF, MCCC and DFQ on frailty in the subgroups, and found that daily MCCC decreases frailty risk in all subgroups. Daily IWF decreases frailty risk in those aged < 65 years, female and rural respondents, but not in those aged ≥ 65 years, male and urban respondents. The effects of occasional and daily MCCC were significant in those aged < 65 years and female participants, whereas only daily MCCC was significant for those aged ≥ 65 years and male participants. The impact of daily DFQ was observed in those aged < 65 years, female and urban respondents, but not in those aged ≥ 65 years, male and rural respondents. Table 6 presents the results in detail.

**Table 6:** Subgroup analysis based on time-varying Cox regression

	Aged ≥ 65 in 2011 (*n *= 1013)^a^	Aged < 65 in 2011 (*n* = 5060)^a^	Male (*n* = 3233)^b^	Female (*n* = 2840)^b^	Rural (*n *= 3653)^c^	Urban (*n* = 2420)^c^
**SP**						
Occasional	0.92 (0.75, 1.11)	0.93 (0.82, 1.06)	0.89 (0.76, 1.04)	0.97 (0.84, 1.13)	0.98 (0.86, 1.11)	0.83 (0.68, 1.02)
Weekly	0.96 (0.77, 1.19)	0.97 (0.84, 1.12)	1.02 (0.86, 1.21)	0.91 (0.77, 1.08)	1.06 (0.91, 1.22)	0.80 (0.64, 1.00)*
Daily	0.76 (0.65, 0.89)**	0.76 (0.68, 0.86)***	0.75 (0.65, 0.87)***	0.77 (0.68, 0.87)***	0.77 (0.68, 0.87)***	0.74 (0.63, 0.86)***
**IWF**						
Occasional	1.10 (0.89, 1.35)	0.90 (0.78, 1.04)	0.90 (0.76, 1.08)	1.01 (0.86, 1.17)	1.05 (0.91, 1.22)	0.81 (0.66, 0.99)*
Weekly	1.08 (0.85, 1.38)	0.95 (0.81, 1.12)	1.13 (0.94, 1.37)	0.86 (0.71, 1.04)	1.10 (0.94, 1.30)	0.80 (0.63, 1.03)
Daily	0.93 (0.78, 1.11)	0.87 (0.76, 1.00)*	0.90 (0.76, 1.07)	0.87 (0.76, 1.00)	0.86 (0.75, 0.98)*	0.94 (0.79, 1.12)
**MCCC**						
Occasional	0.86 (0.66, 1.13)	0.85 (0.72, 1.00)*	0.92 (0.76, 1.11)	0.80 (0.65, 0.98)*	0.90 (0.76, 1.07)	0.78 (0.61, 1.00)
Weekly	0.90 (0.68, 1.17)	0.84 (0.70, 1.02)	0.86 (0.70, 1.06)	0.87 (0.70, 1.09)	0.98 (0.81, 1.18)	0.70 (0.54, 0.91)**
Daily	0.71 (0.55, 0.92)*	0.65 (0.53, 0.81)***	0.68 (0.54, 0.87)**	0.68 (0.54, 0.85)**	0.66 (0.53, 0.82)***	0.68 (0.53, 0.87)**
**DFQ**						
Occasional	0.40 (0.12, 1.27)	0.89 (0.63, 1.28)	0.85 (0.44, 1.67)	0.78 (0.52, 1.16)	0.58 (0.33, 1.00)	1.05 (0.67, 1.63)
Weekly	0.56 (0.21, 1.48)	0.58 (0.35, 0.97)*	0.52 (0.21, 1.24)	0.62 (0.37, 1.05)	0.49 (0.23, 1.03)	0.65 (0.37, 1.16)
Daily	0.89 (0.66, 1.19)	0.63 (0.49, 0.79)***	0.74 (0.54, 1.02)	0.72 (0.57, 0.90)***	0.75 (0.55, 1.01)	0.71 (0.56, 0.90)***

^*^P < 0.05; **P < 0.01; ***P < 0.001. *SP* Social participation, *IWF* Interacting with friends, *MCCC* Playing mah-jong, chess, cards or visiting community clubs, *DFQ* Going to community-organized dancing, fitness, qigong and so on, *HR* Hazard ratio, *CI* Confidence Interval

^a^ The frequency of each type of SP was set as time-variant exposure. ^“^None” group was set as the reference. Age, marital status, hukou status, public health insurance coverage, current work status, alcohol intake, smoking status and household per capita consumption were controlled as time-variant covariates, and gender, education level and residence were controlled as fixed covariates

^b^ All the covariates were controlled except for gender

^c^ All the covariates were controlled except for rural/urban residence

## Discussion

To the best of our knowledge, this study is the first to assess the impact of SP on frailty from the perspectives of frequency and type. The result indicates that, SP, especially a high-frequency one, can significantly decrease the risk of frailty among the middle-aged and older populations. For different types of SP, high-frequency of IWF, MCCC and DFQ are effective exhibits a potential in preventing frailty among non-frail populations and improving the frailty status of already-frail populations. In addition, moderate-frequency of MCCC and DFQ are also associated with decreased risk of frailty.

The prevalence of frailty ranges from 35.5% to 53.5%, which increase with the increase in age. A recent meta-analysis reveals a pooled prevalence of frailty as 10% in China, which is much lower than our study [45]. This could be explained by the measurement of frailty. For example, a study taken into their calculation of frailty prevalence, which also used the CHARLS data, found 7.0% of adults aged 60 years or older were frail [46]. Frailty in that study was measured by the physical frailty phenotype (PFP) scale in which five elements are included: weakness, slowness, exhaustion, inactivity, and shrinking. Some studies indicated that the accumulation of deficits model, as in this study, usually come up with higher percentages of frail than the phenotype measure, as the accumulation of deficits model includes other dimensions of frailty [31]. Chronic diseases and depression were measured as risk factors in Wu’s study [46], nevertheless, which were defined as deficits to calculate frailty score in our analysis. In this study, the prevalences of depressive symptoms are high (almost half of the respondents), and prevalences of chronic diseases, such as hypertension, arthritis and stomach/digestive disease (25.3%, 36.6% and 24.1% at baseline, respectively) are also common. These could further explain the high prevalence of frailty in our study.

The previous literature has suggested that participating in social activities may incentivize mutual support, provide one with a sense of belonging, and largely reduce social isolation, which, therefore, may improve mental health or prevent depression [47]. Furthermore, studies have demonstrated that SP is associated with better cognitive function and lower risk of dementia [48, 49]. Other studies also pointed to the positive effect of SP on physical function [50]. These results indicate the potential association between SP and frailty, where the current study provides a clear evidence for this association.

In this study, SP refers to communicative activities (i.e., interaction with friends), intellectually demanding/engaging activities (i.e., playing mah-jong, chess, and cards or visiting other community clubs), public welfare activities (e.g., participating in community-related organizations and voluntary or charitable work), several community-organized physical activities (going to dancing, fitness, qigong and so on), and online activities (using the Internet). Our analysis indicated that communicative activities, intellectually demanding/engaging activities and community-organized physical activities are more effective in preventing frailty of older population than other SP types. High-frequency communicative activities, such as interacting with friends, can help the older adults to build social networks and increase the opportunities to form intimate relationships, thus helping them to improve social connection and reduce loneliness [51]. In addition, communicative activities are proved to be significantly associated with social network support and effective in decreasing the risk of social isolation and depression [52], consequently decreasing frailty risk. The effectiveness of intellectually demanding/engaging activities, which is observed in all the subgroups, may due to their functions on mental-exercise. As mental-exercise hypothesis indicates, continuous mental cognitive training can prevent individual cognitive decline [53]. Therefore, intellectually demanding/engaging activities can stimulate intellect, and, consequently, maintain cognitive function and prevent frailty among older adults [54]. Daily physical activities, such as exercising or dancing, are also significantly associated with lowered risk of frailty. This result, consistent with those of previous studies [23, 55, 56], indicates that moderate or high-frequency physical activities may be associated with the reduced incidence of disabilities, reduced progression of disabilities, and improved physical functioning, which are associated with lower frailty risk. In addition, we found high frequency of public welfare activities are associated with reversing frailty progression. This can be explained that volunteer activities emphasize individual performance and team cohesion, which are also proved to be more likely to weaken the mortality of older population [57].

Interestingly, the impacts of SP frequency, as well as type, vary among subgroups. For example, the impact of daily IWF, occasional MCCC, and weekly DFQ is significant for those aged < 65 years but not for those aged ≥ 65 years. This difference may be due to the intrinsic property or the irreversible trend of high levels of frailty among older adults aged ≥ 65 years and above, which will be increasingly difficult to control. In addition, the differences in the results for adults in rural and urban areas may be due to the variance in social resources and the stability of social networks. For example, daily IWF is significant in rural but not urban residents, whereas occasional IWF is significant in urban but not rural residents. The potential underlying reasons may be that the social network among rural residents is limited but strong and stable. Nevertheless, the social network among urban residents is relatively weaken. Due to sensitivity from a perception of non-existence to existence for urban respondents, their frailty status is more sensitive to occasional IWF than the rural respondents [22, 58]. Furthermore, variances also existed between male and female respondents. Specifically, female respondents are more sensitive to daily IWF, occasional MCCC, and daily DFQ, which may be due to gender differences in terms of personality, preferences for social interaction, and internal frailty risks [59, 60].

This study provided evidence of the negative association between SP and frailty, which has several implications for healthy ageing strategies. This study employed a nationwide representative database and employed high-quality microdata among middle-aged and older populations. Furthermore, it utilized cohort analysis and different statistical models and comprehensive subgroup analysis. The results suggested other accurate intervention strategies and directions for further prospective mechanism research. Encourageing SP, building esthetic, walkable, and cohesive neighborhood, and providing additional materials and organizational bases for diversified social activities are urgent initiatives for decreasing the risk of frailty for the older population [61].

This study has several limitations. First, the inherent limitations of a retrospective study indicate that the relationship between SP and frailty requires a definitive validation in prospective studies. Second, the study acknowledges the existence of recall bias, because information was self-reported. For example, the indexes associated with physical function and mental health were the main constituent elements of frailty. However, a self-rated level may differ from that of reality. Third, respondents who became frail but died before 2018 have not been included our analysis. Given frailty is associated with mortality, excluding them may introduce a survival bias. Finally, the frailty index covers numerous indicators. During the interview process, many indicators are observed missing among the participants, which led to sample loss. Nevertheless, we applied the cohort design and conducted several sensitivity analyses to overcome this bias.

## Conclusions

In summary, this study used the longitudinal and cohort designs with the fixed-effect and time-varying Cox models to validate the results. The findings clearly demonstrated that enhancing participation in social activities, especially high-frequency SP, could significantly decrease the risk of frailty, as well as reverse frailty progression, among middle-aged and older populations. Furthermore, we identified that communicative activities, intellectually demanding/engaging activities and community-organized physical activities are significantly associated with decreased frailty risk. The impact of the frequency and type of SP vary across age, gender, and residence groups. Thus, these findings present important implications for research and public health policy. As the trend of population ageing has become a worldwide challenge, the current study provided extremely operable, easy, and valuable intervening measures to promote healthy ageing among for middle-aged and older populations around the world.

## Supplementary Information

**Additional file 1.** 

## Data Availability

All the original data could be obtained from the official website of CHARLS (http://charls.pku.edu.cn/). The analysis dataset is available to other researchers and others upon request by emailing the corresponding author (lihaomiao@whu.edu.cn).

## Abbreviations

SP: Social participation
CHARLS: China Health and Retirement Longitudinal Study
OLS: Ordinary least squares
MI: Multiple imputation
FI: Frailty index
IWF: Interacting with friends
MCCC: Playing mah-jong, chess, cards or visiting other community clubs
DFQ: Going to (community-organized) dancing, fitness, qigong and so on
CRO: Participating in community-related organizations
VOC: Voluntary or charitable work
INT: Using the Internet
HR: Hazard ratio
CI: Confidence Interval
PFP: Physical frailty phenotype
