# Supplementary material for: Does social participation decrease the risk of frailty? Impacts of diversity in frequency and types of social participation on frailty in middle-aged and older populations
Source: BMC Geriatr. 2022 Jul 2;22:553. doi: 10.1186/s12877-022-03219-9 (PMC9250233; doi:10.1186/s12877-022-03219-9)
Supplement: Supplementary file 1 — Additional file 1. [file 12877_2022_3219_MOESM1_ESM.docx]

Does social participation decrease the risk of frailty? Impacts of diversity in frequency and types of social participation on frailty in middle-aged and older populations

**Appendix:**

**Table S1** Selection of items to calculate frailty score

**Table S2** Distribution of different SP types across survey waves

**Table S3** Variable description

**Table S4** Data summary before and after multiple imputation

**Table S5** Distribution and prevalence of frailty across survey waves among different SP groups

**Table S6** Baseline description of respondents not frail at baseline

**Table S7** Baseline description of respondents frail at baseline

**Table S1** Selection of items to calculate frailty score*

| Items | Baseline prevalence (%) | Categorization |
| --- | --- | --- |
| Ⅰ. Physical/ADL/IADL limitations (n=18) |  |  |
| Difficulty with walking 100 Metres. | 1.1 | 0=did not have any problems with the activity; 1=some difficulty with the activity or could not do the activity. |
| Difficulty with controlling urination and defecation. | 3.3 |  |
| Difficulty with getting up from chair after sitting for long periods. | 26.7 |  |
| Difficulty with climbing several flights of stairs without resting. | 38.7 |  |
| Difficulty with stooping, kneeling,or crouching. | 28.5 |  |
| Difficulty with reaching or extending arms above shoulder level. | 8.8 |  |
| Difficulty with lifting or carrying weights over 10 Jin (5kg). | 8.7 |  |
| Difficulty with picking up a small coin from a table. | 2.7 |  |
| Difficulty with dressing. | 3.7 |  |
| Difficulty with bathing or showering. | 4.6 |  |
| Difficulty with eating. | 1.8 |  |
| Difficulty with getting out of bed and walking. | 4.1 |  |
| Difficulty with using the toilet, including getting up or down. | 10.2 |  |
| Difficulty with managing money. | 10.1 |  |
| Difficulty with taking medications. | 5.4 |  |
| Difficulty with shopping for groceries. | 6.2 |  |
| Difficulty with preparing a hot meal. | 6.0 |  |
| Difficulty with cleaning house. | 6.5 |  |
| Ⅱ. CES-D (n=10) |  |  |
| Felt depressed much of the time during the past week. | 54.4 | 0=Rarely or none of the time; 1= Some or a little of the time/ Occasionally or a moderate amount of the time/ Most or all of the time. |
| Felt sleep was restless. | 51.1 |  |
| Was not happy most of the time. | 57.2 |  |
| Felt lonely. | 28.3 |  |
| Bothered by little things. | 55.5 |  |
| Could not get going. | 20.7 |  |
| Had trouble keeping mind on what is doing. | 49.2 |  |
| Felt fearful. | 20.4 |  |
| Felt everything they did during the past week was an effort. | 51.5 | 0=Most or all of the time; 1=Some or a little of the time/ Occasionally or a moderate amount of the time/ Rarely or none of the time. |
| Felt hopeful about the future. | 55.9 |  |
| Ⅲ. Comorbidities (n=13) |  |  |
| Hypertension. | 25.3 | 0=never diagnosed; 1=ever diagnosed. |
| Diabetes. | 6.0 |  |
| Lung disease. | 10.0 |  |
| Heart problem. | 12.2 |  |
| Stroke. | 2.1 |  |
| Psych problem. | 1.1 |  |
| Arthritis. | 36.6 |  |
| Dyslipidemia. | 9.8 |  |
| Liver disease. | 3.9 |  |
| Kidney disease. | 6.1 |  |
| Stomach/digestive disease. | 24.1 |  |
| Asthma. | 4.6 |  |
| Memory problem. | 1.3 |  |
| Ⅳ. History of trauma (n=2) |  |  |
| Fractured hip. | 1.5 | 0=never experienced; 1=ever experienced. |
| Fallen down. | 15.5 |  |
| Ⅴ. Cognition (n=7) |  |  |
| Cannot answer correct day of the month given. | 12.3 | 0=can correctly answer; 1=cannot correctly answer. |
| Cannot answer correct month given. | 24.1 |  |
| Cannot answer correct year given. | 25.4 |  |
| Cannot answer correct day given. | 41.3 |  |
| Numeracy. | 59.5 |  |
| Immediate recall. | 56.3 | 0=could correctly recall at least 6 words; 1=could not correctly recall at least 6 words. |
| Delayed recall. | 52.1 | 0=could correctly recall at least 7 words; 1=could not correctly recall at least 7 words. |
| Ⅵ. Others (n=4) |  |  |
| Hearing problem. | 6.6 | 0=never had; 1=ever had. |
| Eyesight problem. | 5.5 |  |
| Self reported pain. | 33.9 | 0=never troubled with any body pains; 1=a little/ somewhat/ quite a bit/ very often troubled with body pains. |
| Self reported health. | 74.8 | 0=very good/ good; 1=fair/ poor/ very poor. |

*The frailty index was calculated by summing the number of deficits reported by the respondents and dividing by the total number of possible (answered) deficits. For the chronic disease variables (e.g., hypertension), a score of 1 in one wave was allocated in all subsequent waves as the conditions were irreversible. Then, a frailty index with a potential range from 0 to 1 was generated, with a higher score indicating more serious frailty.

**Table S2** Distribution of different SP types across survey waves

| WAVE | 2011 | 2013 | 2015 | 2018 | *P*-value* |
| --- | --- | --- | --- | --- | --- |
| IWF |  |  |  |  | <0.001 |
| none | 6014 (63.83%) | 5565 (59.06%) | 6089 (64.63%) | 6415 (68.09%) |  |
| not regular | 1008 (10.70%) | 1251 (13.28%) | 1215 (12.90%) | 1030 (10.93%) |  |
| weekly | 741 (7.86%) | 819 (8.69%) | 700 (7.43%) | 672 (7.13%) |  |
| daily | 1659 (17.61%) | 1787 (18.97%) | 1418 (15.05%) | 1305 (13.85%) |  |
| MCCC |  |  |  |  | <0.001 |
| none | 7645 (81.14%) | 7397 (78.51%) | 7583 (80.48%) | 7874 (83.57%) |  |
| not regular | 767 (8.14%) | 788 (8.36%) | 725 (7.69%) | 556 (5.90%) |  |
| weekly | 578 (6.13%) | 646 (6.86%) | 565 (6.00%) | 473 (5.02%) |  |
| daily | 432 (4.59%) | 591 (6.27%) | 549 (5.83%) | 519 (5.51%) |  |
| DFQ |  |  |  |  | <0.001 |
| none | 8915 (94.62%) | 8678 (92.10%) | 8692 (92.25%) | 8896 (94.42%) |  |
| not regular | 89 (0.94%) | 123 (1.31%) | 149 (1.58%) | 86 (0.91%) |  |
| weekly | 96 (1.02%) | 107 (1.14%) | 87 (0.92%) | 88 (0.93%) |  |
| daily | 322 (3.42%) | 514 (5.46%) | 494 (5.24%) | 352 (3.74%) |  |
| CRO |  |  |  |  | <0.001 |
| none | 9291 (98.61%) | 9226 (97.92%) | 9196 (97.60%) | 9225 (97.91%) |  |
| not regular | 73 (0.77%) | 107 (1.14%) | 144 (1.53%) | 114 (1.21%) |  |
| weekly | 44 (0.47%) | 65 (0.69%) | 61 (0.65%) | 58 (0.62%) |  |
| daily | 14 (0.15%) | 24 (0.25%) | 21 (0.22%) | 25 (0.27%) |  |
| VOC |  |  |  |  | <0.001 |
| none | 8628 (91.57%) | 7982 (84.72%) | 7779 (82.56%) | 8089 (85.85%) |  |
| not regular | 560 (5.94%) | 1013 (10.75%) | 1209 (12.83%) | 902 (9.57%) |  |
| weekly | 147 (1.56%) | 286 (3.04%) | 287 (3.05%) | 268 (2.84%) |  |
| daily | 87 (0.92%) | 141 (1.50%) | 147 (1.56%) | 163 (1.73%) |  |
| INT |  |  |  |  | <0.001 |
| none | 9235 (98.02%) | 9131 (96.91%) | 9063 (96.19%) | 8661 (91.92%) |  |
| not regular | 41 (0.44%) | 44 (0.47%) | 57 (0.60%) | 80 (0.85%) |  |
| weekly | 32 (0.34%) | 49 (0.52%) | 39 (0.41%) | 56 (0.59%) |  |
| daily | 114 (1.21%) | 198 (2.10%) | 263 (2.79%) | 625 (6.63%) |  |

SP, social participation; IWF, interacting with friends; MCCC, playing mah-jong, chess, cards or visiting community clubs; DFQ, going to community-organized dancing, fitness, qigong and so on; CRO, participating in community-related organizations; VOC, voluntary or charitable work; INT, using the Internet.

**P* values were calculated by Chi-square test, to assess the distribution variance of frequency in each SP type across the survey waves.

**Table S3** Variable description

| **Variable name** | **Definition or code** |
| --- | --- |
| **Outcome** |  |
| Frailty | 0=non-frail or pre-frail (frailty score<0.25); 1=frail (frailty score ≥ 0.25 to 1.00). |
| **Exposure** |  |
| Social participation (SP) | SP was measured with frequency and type.  Frequency: (1) none, (2) occasional, (3) weekly, and (4) daily.  Type: (1) interacting with friends (IWF), (2) playing mah-jong, chess, and cards or visiting community clubs (MCCC), (3) going to community-organized dancing, fitness, qigong and so on (DFQ), (4) participating in community-related organizations (CRO), (5) voluntary or charitable work, and (6) using the Internet (INT).  The frequency of each SP type is consistent with the comprehensive SP (none; occasional; weekly; daily). |
| **Covariates** |  |
| Age | -- |
| Gender | 1=male; 2=female. |
| Marital status | 1=married or partnered; 2=separated, divorced or widowed. |
| Hukou status | Hukou indicates the respondent’s hukou place and is a special identifier in China. Hukou status affects many aspects of life in China such as buying a house, buying a car, children’s school enrollment and other welfare. 1=Agricultual hukou; 2=Non-agricultural hukou; 3=Unified residence hukou or do not have hukou. |
| Education levels | Education level is a simplified version of 1997 International Standard Classification of Education (ISCED-97) codes. 1=Less than lower secondary education; 2=Upper secondary &  vocational training; 3=Tertiary education. |
| Rural/urban residence | Residence indicates the household living region and is defined by  National Bureau of Statistics of the People's Republic of China. 1=rural; 2=urban. |
| Public health insurance coverage | A code of 0 indicates that the respondent is not covered by any public health insurance plan. A code of 1 indicates that the respondent is covered by at least one type of public health insurance plan, including Urban Employee Medical Insurance, Urban Resident Medical Insurance, New Cooperative Medical Insurance, Urban and Rural Resident Medical Insurance, Government Medical Insurance, Medical Aid or other government insurance plan. |
| Current work status | Current work status indicates whether the respondent engaged in any work in the past year. Participants were coded as 1 if they engaged in agricultural work, non-agricultural employed work, non-agricultural  self-employment work, or non-agricultural family business work per the labor force status of each wave respectively. And this variable was assigned 0 if the respondent is unemployed, retired, or never worked  per the labor force status of each wave respectively. |
| Alcohol intake | Alcohol intake indicates whether the respondent has had an alcoholic beverage in the last 12 months (obtained from the CHARLS database). A code of 0 indicates that the respondent reports not having any alcoholic beverage in the last 12  months. A code of 1 indicates that the respondent reports having had an alcoholic beverage in the last 12 months. |
| Smoking status | It indicates the respondent’s current smoking habit. 0=never smoke; 1=ever smoke but quit now; 2=still smoking now. |
| Household per capita consumption | Household per capita consumption is calculated by taking total  household consumption divided by the number of people in the household. The amount of total household consumption as aggregated from all consumption activities: food consumption in last week, non-food in the past 30 days, and other non-food consumption in the past year. The household per capita consumptions in different survey waves were adjusted by the Consumer Price Index, and then divided into four groups based on quartile. The details: (1)Low: <¥4502.3; (2)Low to middle: ≥¥4502.3 and <¥8225.3; (3)Middle: ≥¥8225.3 and <¥15199.5; (4)High: ≥¥15199.5. |

**Table S4** Data summary before and after multiple imputation^*^

|  | Number of missing values | Variable type | Mean before imputation | Mean after imputation |
| --- | --- | --- | --- | --- |
| Age | 0 | continuous | 60.73 | 60.73 |
| Gender | 0 | binary | 1.54 | 1.54 |
| Education level | 0 | ordered-categorical | 1.12 | 1.12 |
| Marital status | 5 | binary | 0.87 | 0.87 |
| Hukou status | 560 | ordered-categorical | 1.20 | 1.20 |
| Public health insurance coverage | 86 | binary | 0.95 | 0.95 |
| Current work status | 104 | binary | 0.70 | 0.70 |
| Comorbidity | 0 | ordered-categorical | 1.33 | 1.33 |
| Household per capita consumption group | 7068 | ordered-categorical | 2.50 | 2.49 |
| Alcohol intake | 15 | binary | 0.33 | 0.33 |
| Smoking status | 2179 | ordered-categorical | 0.63 | 0.66 |
| Rural/urban residence | 0 | binary | 1.35 | 1.35 |

^*^ The imputing method was from the posterior predictive distribution (ppd).

**Table S5** Distribution and prevalence of frailty across survey waves among different SP groups

|  | 2011 | | 2013 | | 2015 | | 2018 | |
| --- | --- | --- | --- | --- | --- | --- | --- | --- |
|  | Mean±SD | Prevalence | Mean±SD | Prevalence | Mean±SD | Prevalence | Mean±SD | Prevalence |
| Non-SP | 0.23 ± 0.12 | 1864 (39.94%) | 0.24 ± 0.12 | 1616 (40.26%) | 0.26 ± 0.14 | 2015 (45.36%) | 0.29 ± 0.14 | 2708 (57.03%) |
| Occasional | 0.22 ± 0.12 | 513 (36.36%) | 0.22 ± 0.12 | 551 (35.34%) | 0.24 ± 0.13 | 643 (40.14%) | 0.27 ± 0.13 | 682 (52.22%) |
| Weekly | 0.20 ± 0.11 | 329 (29.88%) | 0.21 ± 0.11 | 358 (30.65%) | 0.23 ± 0.13 | 384 (37.65%) | 0.27 ± 0.13 | 516 (55.25%) |
| Daily | 0.20 ± 0.12 | 643 (28.67%) | 0.20 ± 0.11 | 781 (29.13%) | 0.22 ± 0.13 | 802 (34.01%) | 0.25 ± 0.13 | 1135 (46.63%) |
| All | 0.22 ± 0.12 | 3349 (35.54%) | 0.22 ± 0.12 | 3306 (35.09%) | 0.24 ± 0.13 | 3844 (40.80%) | 0.28 ± 0.14 | 5041 (53.50%) |

SP, social participation; SD, standard deviation.

*Mean±standard deviation was used to describe continuous variables, and number (constituent ratio [%]) was used to describe categorical variables.

**Table S6** Baseline description of respondents not frail at baseline

|  | All | Non-SP | Occasional | Weekly | Daily |
| --- | --- | --- | --- | --- | --- |
| Number of participants | 6073 | 2803 | 898 | 772 | 1600 |
| Age | 56.65 ± 8.08 | 56.85 ± 7.90 | 55.44 ± 7.65 | 55.79 ± 8.02 | 57.41 ± 8.55 |
| Gender |  |  |  |  |  |
| Male | 3233 (53.24%) | 1495 (53.34%) | 532 (59.24%) | 459 (59.46%) | 747 (46.69%) |
| Female | 2840 (46.76%) | 1308 (46.66%) | 366 (40.76%) | 313 (40.54%) | 853 (53.31%) |
| Education levels |  |  |  |  |  |
| Less than lower secondary | 5169 (85.11%) | 2505 (89.37%) | 750 (83.52%) | 626 (81.09%) | 1288 (80.50%) |
| Upper secondary & vocational training | 789 (12.99%) | 278 (9.92%) | 132 (14.70%) | 126 (16.32%) | 253 (15.81%) |
| Tertiary | 115 (1.89%) | 20 (0.71%) | 16 (1.78%) | 20 (2.59%) | 59 (3.69%) |
| Marital status |  |  |  |  |  |
| Divorced or widowed | 487 (8.02%) | 222 (7.92%) | 60 (6.68%) | 39 (5.05%) | 166 (10.38%) |
| Married | 5586 (91.98%) | 2581 (92.08%) | 838 (93.32%) | 733 (94.95%) | 1434 (89.62%) |
| Hukou status |  |  |  |  |  |
| Agricultual | 4732 (77.93%) | 2340 (83.51%) | 711 (79.18%) | 594 (76.94%) | 1087 (67.94%) |
| Non-agricultural | 1293 (21.29%) | 440 (15.70%) | 182 (20.27%) | 172 (22.28%) | 499 (31.19%) |
| Other | 47 (0.77%) | 22 (0.79%) | 5 (0.56%) | 6 (0.78%) | 14 (0.88%) |
| Rural/urban residence |  |  |  |  |  |
| Rual | 3653 (60.15%) | 1789 (63.82%) | 565 (62.92%) | 467 (60.49%) | 832 (52.00%) |
| Urban | 2420 (39.85%) | 1014 (36.18%) | 333 (37.08%) | 305 (39.51%) | 768 (48.00%) |
| Morbidity |  |  |  |  |  |
| None | 2441 (40.19%) | 1154 (41.17%) | 379 (42.20%) | 302 (39.12%) | 606 (37.88%) |
| Single | 1985 (32.69%) | 948 (33.82%) | 295 (32.85%) | 241 (31.22%) | 501 (31.31%) |
| Morbidity | 1647 (27.12%) | 701 (25.01%) | 224 (24.94%) | 229 (29.66%) | 493 (30.81%) |
| Public health insurance coverage |  |  |  |  |  |
| Not covered | 380 (6.27%) | 178 (6.36%) | 49 (5.46%) | 41 (5.32%) | 112 (7.01%) |
| Covered | 5683 (93.73%) | 2620 (93.64%) | 848 (94.54%) | 730 (94.68%) | 1485 (92.99%) |
| Current work status |  |  |  |  |  |
| Not working | 1364 (22.51%) | 509 (18.20%) | 143 (15.96%) | 160 (20.78%) | 552 (34.59%) |
| Working | 4695 (77.49%) | 2288 (81.80%) | 753 (84.04%) | 610 (79.22%) | 1044 (65.41%) |
| Alcohol intake |  |  |  |  |  |
| Do not drink | 3809 (62.72%) | 1831 (65.32%) | 505 (56.24%) | 429 (55.57%) | 1044 (65.25%) |
| Drink | 2264 (37.28%) | 972 (34.68%) | 393 (43.76%) | 343 (44.43%) | 556 (34.75%) |
| Smoking status |  |  |  |  |  |
| Never | 3519 (57.95%) | 1652 (58.96%) | 465 (51.78%) | 405 (52.46%) | 997 (62.31%) |
| Quit now | 489 (8.05%) | 213 (7.60%) | 86 (9.58%) | 77 (9.97%) | 113 (7.06%) |
| Smoke | 2064 (33.99%) | 937 (33.44%) | 347 (38.64%) | 290 (37.56%) | 490 (30.63%) |
| Household per capita consumption |  |  |  |  |  |
| Low | 1896 (36.31%) | 953 (39.96%) | 300 (38.22%) | 219 (33.38%) | 424 (30.37%) |
| Low to middle | 1518 (29.07%) | 720 (30.19%) | 233 (29.68%) | 179 (27.29%) | 386 (27.65%) |
| Middle | 1180 (22.60%) | 499 (20.92%) | 166 (21.15%) | 171 (26.07%) | 344 (24.64%) |
| High | 628 (12.03%) | 213 (8.93%) | 86 (10.96%) | 87 (13.26%) | 242 (17.34%) |

SP, social participation. Mean±standard deviation was used to describe continuous variables, and number (constituent ratio [%]) was used to describe categorical variables.

**Table S7** Baseline description of respondents frail at baseline

|  | All | Non-SP | Occasional | Weekly | Daily |
| --- | --- | --- | --- | --- | --- |
| Number of participants | 3349 | 1864 | 513 | 329 | 643 |
| Age | 59.55 ± 8.20 | 59.87 ± 7.95 | 58.18 ± 8.14 | 58.91 ± 8.68 | 60.07 ± 8.58 |
| Gender |  |  |  |  |  |
| Male | 1129 (33.71%) | 635 (34.07%) | 182 (35.48%) | 127 (38.60%) | 185 (28.77%) |
| Female | 2220 (66.29%) | 1229 (65.93%) | 331 (64.52%) | 202 (61.40%) | 458 (71.23%) |
| Education levels |  |  |  |  |  |
| Less than lower secondary | 3193 (95.34%) | 1793 (96.19%) | 489 (95.32%) | 308 (93.62%) | 603 (93.78%) |
| Upper secondary & vocational training | 144 (4.30%) | 66 (3.54%) | 22 (4.29%) | 20 (6.08%) | 36 (5.60%) |
| Tertiary | 12 (0.36%) | 5 (0.27%) | 2 (0.39%) | 1 (0.30%) | 4 (0.62%) |
| Marital status |  |  |  |  |  |
| Divorced or widowed | 464 (13.85%) | 254 (13.63%) | 60 (11.70%) | 50 (15.20%) | 100 (15.55%) |
| Married | 2885 (86.15%) | 1610 (86.37%) | 453 (88.30%) | 279 (84.80%) | 543 (84.45%) |
| Hukou status |  |  |  |  |  |
| Agricultual | 2979 (88.95%) | 1669 (89.54%) | 471 (91.81%) | 286 (86.93%) | 553 (86.00%) |
| Non-agricultural | 354 (10.57%) | 185 (9.92%) | 41 (7.99%) | 41 (12.46%) | 87 (13.53%) |
| Other | 16 (0.48%) | 10 (0.54%) | 1 (0.19%) | 2 (0.61%) | 3 (0.47%) |
| Rural/urban residence |  |  |  |  |  |
| Rual | 2500 (74.65%) | 1371 (73.55%) | 409 (79.73%) | 250 (75.99%) | 470 (73.09%) |
| Urban | 849 (25.35%) | 493 (26.45%) | 104 (20.27%) | 79 (24.01%) | 173 (26.91%) |
| Morbidity |  |  |  |  |  |
| None | 402 (12.00%) | 243 (13.04%) | 52 (10.14%) | 35 (10.64%) | 72 (11.20%) |
| Single | 820 (24.48%) | 471 (25.27%) | 147 (28.65%) | 68 (20.67%) | 134 (20.84%) |
| Morbidity | 2127 (63.51%) | 1150 (61.70%) | 314 (61.21%) | 226 (68.69%) | 437 (67.96%) |
| Public health insurance coverage |  |  |  |  |  |
| Not covered | 186 (5.57%) | 111 (5.97%) | 20 (3.91%) | 16 (4.86%) | 39 (6.11%) |
| Covered | 3151 (94.43%) | 1747 (94.03%) | 492 (96.09%) | 313 (95.14%) | 599 (93.89%) |
| Current work status |  |  |  |  |  |
| Not working | 955 (28.61%) | 527 (28.41%) | 109 (21.25%) | 82 (25.00%) | 237 (36.92%) |
| Working | 2383 (71.39%) | 1328 (71.59%) | 404 (78.75%) | 246 (75.00%) | 405 (63.08%) |
| Alcohol intake |  |  |  |  |  |
| Do not drink | 2499 (74.62%) | 1415 (75.91%) | 353 (68.81%) | 239 (72.64%) | 492 (76.52%) |
| Drink | 850 (25.38%) | 449 (24.09%) | 160 (31.19%) | 90 (27.36%) | 151 (23.48%) |
| Smoking status |  |  |  |  |  |
| Never | 2291 (68.41%) | 1287 (69.05%) | 329 (64.13%) | 215 (65.35%) | 460 (71.54%) |
| Quit now | 263 (7.85%) | 149 (7.99%) | 38 (7.41%) | 27 (8.21%) | 49 (7.62%) |
| Smoke | 795 (23.74%) | 428 (22.96%) | 146 (28.46%) | 87 (26.44%) | 134 (20.84%) |
| Household per capita consumption |  |  |  |  |  |
| Low | 1282 (44.70%) | 746 (46.83%) | 189 (42.57%) | 125 (42.23%) | 222 (41.50%) |
| Low to middle | 851 (29.67%) | 472 (29.63%) | 130 (29.28%) | 80 (27.03%) | 169 (31.59%) |
| Middle | 526 (18.34%) | 268 (16.82%) | 94 (21.17%) | 59 (19.93%) | 105 (19.63%) |
| High | 209 (7.29%) | 107 (6.72%) | 31 (6.98%) | 32 (10.81%) | 39 (7.29%) |

SP, social participation. Mean±standard deviation was used to describe continuous variables, and number (constituent ratio [%]) was used to describe categorical variables.
